# Knockdown of *OLR1* weakens glycolytic metabolism to repress colon cancer cell proliferation and chemoresistance by downregulating SULT2B1 via c-MYC

**DOI:** 10.1038/s41419-021-04174-w

**Published:** 2021-12-17

**Authors:** Tiancheng Zhao, Yezhou Li, Kexin Shen, Quan Wang, Jiayu Zhang

**Affiliations:** 1 Department of Endoscopy Center, China-Japan Union Hospital of Jilin University, Changchun, 130033 P.R. China; 2 Department of Vascular Surgery, China-Japan Union Hospital of Jilin University, Changchun, 130033 P.R. China; 3 Gastrointestinal Colorectal and Anal Surgery, China-Japan Union Hospital of Jilin University, Changchun, 130033 P.R. China; 4 Department of Radiation Oncology, China-Japan Union Hospital of Jilin University, Changchun, 130033 P.R. China

**Keywords:** Cancer, Cancer therapy

## Abstract

Chemoresistance is one of the major problems of colon cancer treatment. In tumors, glycolytic metabolism has been identified to promote cell proliferation and chemoresistance. However, the molecular mechanisms underlying glycolytic metabolism and chemoresistance in colon cancer remains enigmatic. Hence, this research was designed to explore the mechanism underlying the *OLR1*/c-MYC/SULT2B1 axis in the regulation of glycolytic metabolism, to affect colon cancer cell proliferation and chemoresistance. Colon cancer tissues and LoVo cells were attained, where *OLR1*, c-MYC, and SULT2B1 expression was detected by immunohistochemistry, RT-qPCR, and western blot analysis. Next, ectopic expression and knockdown assays were implemented in LoVo cells. Cell proliferation was detected by MTS assay and clone formation. Extracellular acidification, glucose uptake, lactate production, ATP/ADP ratio, and GLUT1 and LDHA expression were measured to evaluate glycolytic metabolism. Then, the transfected cells were treated with chemotherapeutic agents to assess drug resistance by MTS experiments and P-gp and SMAD4 expression by RT-qPCR. A nude mouse model of colon cancer transplantation was constructed for in vivo verification. The levels of *OLR1*, c-MYC, and SULT2B1 were upregulated in colon cancer tissues and cells. Mechanistically, *OLR1* increased c-MYC expression to upregulate SULT2B1 in colon cancer cells. Moreover, knockdown of *OLR1*, c-MYC, or SULT2B1 weakened glycolytic metabolism, proliferation, and chemoresistance of colon cancer cells. In vivo experiments authenticated that *OLR1* knockdown repressed the tumorigenesis and chemoresistance in nude mice by downregulating c-MYC and SULT2B1. Conclusively, knockdown of *OLR1* might diminish SULT2B1 expression by downregulating c-MYC, thereby restraining glycolytic metabolism to inhibit colon cancer cell proliferation and chemoresistance.

## Introduction

Colon cancer initiates with an extensive block of cell replication with replication foci shifting towards the upper parts of crypts [1]. Besides, colon cancer is clarified into three discernible stages: initiation, promotion, and progression, where the normal colonic epithelium pathologically transforms into hyperproliferative epitheliums, then into adenomas and carcinoma in situ, and finally into invasive and metastatic cancer [2]. It is well-known that the risk factor for colon cancer includes red meat intake, dietary factors, age, ethnicity, sex, history of colon cancer, enhanced body mass index (BMI), low vegetable and fruit consumption, genetic makeup, low physical activity, and long-term cigarette smoking (30–40 years) [3]. Up to date, patients with colon cancer are generally treated with radiotherapy, cytotoxic drugs, surgery, antiangiogenic agents, and chemotherapy [4]. During treatment, elevated glycolytic metabolism has been observed to assume a pivotal role in adenosine triphosphate (ATP) source in cancer, and ATP-dependent homeostatic maintenance of resting [Ca2^+^] is crucial in proliferation and chemoresistance of colorectal cancer (CRC) cells [5]. Resistance to chemotherapeutic agents is one of the principal obstacles related to colon cancer treatment, which engenders the need to develop novel therapies’ targets [6].

As a lectin-like scavenger receptor, oxidized low-density lipoprotein receptor 1 (*OLR1*, a gene that encodes for the lectin-like oxidized low-density lipoprotein receptor-1 [LOX-1] protein) recognizes various ligands, including oxidized low-density lipoprotein and has been documented to be involved in cardiovascular and metabolic diseases [7]. Moreover, ectopic expression of *OLR1* has been noted to augment colon cancer onset, progression, and metastasis [8]. More importantly, a prior work indicated the promoting effects of *OLR1* on gemcitabine resistance in pancreatic cancer [9]. Interestingly, it was illustrated in another research that *OLR1* upregulation could enhance c-MYC expression to accelerate pancreatic cancer metastasis [10]. As one of the most critical transcription factors, c-MYC is involved in cancer cells reprogramming, proliferation, and chemoresistance [11]. Existing literature has suggested that ectopically expressed c-MYC facilitates glucose metabolism, viability, and migration of colon cancer cells [12]. Therefore, c-MYC may be implicated in the chemoresistance of colon cancer, which needs to be further verified. Moreover, the overexpression of sulfotransferase (SULT) 2B1 has been exhibited in CRC tissues [13]. Given the aforementioned exploration of literature, we proposed a hypothesis that the network of *OLR1*, c-MYC, and SULT2B1 might orchestrate metastasis and chemoresistance in colon cancer. Therefore, tissue, cell, and animal assays were implemented in our research to confirm this hypothesis, thus providing newer targets for more effect treatment of colon cancer.

## Materials and methods

### Bioinformatics analysis

The expression dataset GSE10950 (normal samples: 24 cases; colon cancer samples: 24 cases), GSE41328 (normal samples: 10 cases; colon cancer samples: 10 cases), and GSE75970 (normal samples: 4 cases; colon cancer samples: 4 cases) in Gene Expression Omnibus (GEO, https://www.ncbi.nlm.nih.gov/gds) database was differentially analyzed by “limma” package of R language (https://bioconductor.org/packages/limma/). The significantly upregulated genes were screened out with logFC > 2 and *p* < 0.05 as the threshold. Significant upregulated genes were obtained from the intersection of the results of three datasets. Totally, 50 genes most related to colon cancer were obtained from MalaCards (https://www.malacards.org/). The protein interaction network between the significantly upregulated genes and colon cancer-related genes was constructed by String (minimum required interaction score: 0.4, https://string-db.org/). The Cytoscape (https://cytoscape.org/) was used to draw protein–protein interaction network and calculate the core degree of significantly upregulated genes.

The Gene Expression Profiling Interactive Analysis (GEPIA, http://gepia2.cancer-pku.cn/#index) was used to analyze the clinical data (COADREAD) of The Cancer Genome Atlas (TCGA, https://portal.gdc.cancer.gov/) database to determine the impact of key genes on the survival rate of patients with colon cancer. The downstream transcription factors of key genes were identified by literature review. The expression data of key genes and downstream transcription factors in GSE10950, GSE41328, and GSE75970 were normalized. Pearson correlation analysis was used to verify the relationship between key genes and downstream transcription factors. The hTFtarget (http://bioinfo.life.hust.edu.cn/hTFtarget#!/) was used to predict the downstream genes of transcription factors and obtain important downstream genes by crossing with important significantly upregulated genes. The GEPIA was adopted to analyze the clinical data (COAD, READ) of TCGA database to identify the genes most related to the survival of colon cancer. The Kyoto Encyclopedia of Genes and Genomes (KEGG, https://www.kegg.jp/kegg/) was used to find the pathways involved in the key downstream genes, and the function of KEGG in colon cancer was predicted based on the literature.

### Clinical samples

From January 2015 to January 2017, 120 patients diagnosed with colon cancer in China-Japan Union Hospital of Jilin University were operated to obtain cancer tissues and normal adjacent tissues. All patients were diagnosed as with colon cancer by pathological diagnosis [14] and did not receive neoadjuvant therapy such as preoperative chemoradiotherapy. Patients meeting the following conditions were excluded from the colon cancer experimental group [1]: advanced cancer with obvious cachexia [2]; multiple tumors [3]; patients with other serious chronic physical diseases, acute infection or liver and kidney failure [4]; patients with mental illness, history of dementia or other reasons cannot communicate; and patients with taking drugs that can cause anxiety and depression.

### Immunohistochemical staining

Paraffin sections were made from colon cancer tissues and adjacent normal tissues. The sections were put into a 60 °C incubator for 1 h and taken out. The sections were dewaxed in xylene I and xylene II for 30 min individually. After the sections were hydrated with gradient alcohol, the sections were put into a beaker containing diluted potassium citrate solution and subjected to 90 °C microwave antigen thermal repair for 10 min. Then the sections were cooled at room temperature. The endogenous peroxidase was inactivated by adding 3% H_2_O_2_ in turn at room temperature for 10 min. The sections were sealed at room temperature for 20 min with 5% goat serum (Beijing Solarbio Science & Technology Co. Ltd., Beijing, China). After discarding the blocking solution, the sections were dripped with monoclonal antibodies (Abcam, Cambridge, MA, USA) to LOX-1 (1:100, ab81709) and c-MYC (1:100, ab32072), and polyclonal antibodies (Abcam) to SULT2B1 (1:50, ab254616) and Ki67 (1:50, ab15580) to completely cover the sections, and incubated at 4 °C overnight. Afterwards, the sections were reprobed for 1 h at 37 °C with goat-anti-rabbit and anti-mouse secondary antibodies (ZSGB-Bio, Beijing, China). The sections were stained with diaminobenzidine (ZSGB-BIO) for 3–5 min. Multi-functional true-color cell image analysis and management system (Media Cybernetics, Silver Spring, MD, USA) was used for analysis. Totally, four sections were taken from each specimen, and three fields of vision were randomly taken from the sections. The percentage of positive cells was counted under Olympus provis (Olympus, Tokyo, Japan) and the average value was taken to reflect the expression of LOX-1, c-MYC, and SULT2B1, respectively.

### Reverse-transcription quantitative polymerase chain reaction (RT-qPCR)

The Trizol (Invitrogen Inc., Carlsbad, CA, USA) was used to extract total RNA from colon cancer tissues and cells. After RNA concentration was measured by Nanodrop 2000 (Thermo Fisher Scientific Inc., Waltham, MA, USA), 1 μg total RNA was reverse transcribed into cDNA by PrimeScript^TM^ RT reagent kit with gDNA Eraser Kit (Takara Holdings Inc., Kyoto, Japan). After adding 5 g DNA Eraser Buffer and gDNA Eraser according to the instructions, DNA elimination reaction was carried out at 42 °C for 2 min. After the reagent was added, the cDNA was obtained by reverse transcription at 37 °C for 15 min and 85 °C for 5 s, and then used for qPCR experiment. Real-time PCR was performed on ABI7500 quantitative PCR instrument (Thermo Fisher Scientific Inc.) with SYBR^®^ Premix Ex Taq^TM^ (Tli RNaseH Plus) Kit (Takara Holdings Inc.). The 2^−ΔΔCT^ was used to express the multiple ratio of target gene expression between the observation group and the reference group with glyceraldehyde-3-phosphate dehydrogenase (GAPDH) as internal reference. The formula was as follows: ΔΔ CT = Δ CT _observation group_ − Δ CT _reference group_, where Δ CT = CT _target gene_ − CT _β-actin_ [15]. The primers used in the reaction were shown in Supplementary Table 1. The primers were provided by Shanghai GenePharma Co. Ltd. (Shanghai, China). The experiment was repeated three times.

### Western blot analysis

The Bicinchoninic Acid Kit (Thermo Fisher Scientific Inc.) was used to detect the protein concentration in colon tumor tissues of nude mice. The 30 μg of total protein was electrophoresed by sodium dodecyl sulfate polyacrylamide gel electrophoresis with constant voltage of 80 V for 35 min, followed by 120 V for 45 min. After electrophoresis, it was transferred to a polyvinylidene fluoride membrane (Amersham Biosciences/GE Healthcare, Piscataway, NJ, USA). The membrane was sealed at room temperature for 1 h with 5% skimmed milk powder. After dumping the sealing fluid, the membrane was probed overnight with antibodies (Abcam) to LOX-1 monoclonal antibody (1:1000, ab214427), c-MYC monoclonal antibody (1:1000, ab32072), SULT2B1 polyclonal antibody (1:500, ab254616), and β-actin monoclonal antibody (1:5000, ab8225) at 4 °C. The membrane was washed with Tris-buffered saline with Tween 20 (TBST) buffer (Tris-buffered saline buffer containing 0.1% Tween-20) for 3 times/10 min. Afterwards, the membrane was reprobed for 1 h with horseradish peroxidase (HRP)-tagged goat-anti-mouse or anti-rabbit (1:10,000, 115–035–003; Jackson Laboratory, Bar Harbor, ME, USA) secondary antibodies at room temperature. The membrane was washed with TBST for 3 times/10 min. After scanning and developing by optical luminescent instrument (General Electric Company, Schenectady, NY, USA), the gray analysis of protein bands was carried out using Image Pro Plus 6.0 software (Media Cybernetics, Silver Spring, MD, USA), and the relative expression of other proteins in corresponding samples was corrected with the amount of β-actin protein as internal reference. The experiment was repeated three times.

### Cell culture and lentivirus transfection

Human colon cancer LoVo cell line was purchased from American Type Culture Collection (ATCC, VA, USA) and cultured in 37 °C, 5% CO_2_ incubator. The *OLR1* knockdown lentivirus, c-MYC overexpression lentivirus, c-MYC knockdown lentivirus, SULT2B1 overexpression lentivirus, and blank vector lentivirus were purchased from Shanghai Genechem Co., Ltd. (Shanghai, China). The cells were infected according to the lentivirus infection manuals provided by Shanghai Genechem Co., Ltd. According to the virus titer multiplicity of infection (MOI) = 5, appropriate amount of lentivirus was added into the cell culture plate. After 48 h of culture, the puromycin (60210ES25, Yeasen Company, Shanghai, China) was added to screen stable cell lines. The expression of *OLR1*, c-MYC, and SULT2B1 in each cell line was detected by RT-qPCR to ensure effective cell lines.

The LoVo cells were infected with lentiviruses harboring: (1) short hairpin (sh)-negative control (NC) or sh-*OLR1*; (2) overexpression (oe)-NC, oe-c-MYC, sh-NC, or sh-c-MYC; (3) sh-NC + oe-NC, sh-*OLR1* + oe-NC, or sh-*OLR1* + oe-c-MYC; (4) sh-NC + oe-NC, sh-c-MYC + oe-NC, or sh-c-MYC + oe-SULT2B1; (5) sh-NC + oe-NC, sh-*OLR1* + oe-NC, or sh-*OLR1* + oe-SULT2B1.

### MTS experiment

Colon cancer cells in logarithmic growth phase were digested with trypsin to prepare 3 × 10^5^ cells/mL cell suspension. The 100 μL cell suspension was added to each well of a 96-well plate. After the cells adhered to the wall, cells were added with 100 μL chemotherapeutic drugs prepared by culture medium containing 10% serum [oxaliplatin (O9512-5MG; the concentration gradients of 0, 2, 4, 8, 16, and 32 mg/L), 5-fluorouracil (5-Fu, F5130-100MG; the concentration gradients of 0, 2.5, 5, 10, 20 and 40 mg/L), cisplatin (DDP, P4394-25MG; the concentration gradients of 0, 0.5, 1, 2, 4, and 8 mg/L), paclitaxel (444375-500MG; the concentration gradients of 0, 12.5, 25, 50, 100, and 200 mg/L), the above drugs were purchased from Sigma-Aldrich Chemical Company (St Louis, MO, USA)] [16]. After 72 h of administration, 20 μL of MTS reagent (G1111; Promega Corporation, Madison, WI, USA) was added to each well and incubated in a 37 °C and 5% CO_2_ incubator for 4 h. The absorbance (*A*) at 490 nm (A490) of each well was detected on the multi-functional microplate reader. The survival rate and inhibition rate were calculated by the absorbance value. According to the linear regression equation of drug concentration and inhibition rate, the IC_50_ value of drug inhibition rate was determined. Cell viability curve was drawn with concentration as abscissa and OD value as ordinate. In addition, the culture plates were taken out severally after 12 h, 24, 36, and 48 h of culture. The 20 μL MTS reagent was added to each well, and the cell proliferation level was detected after 4 h of culture. Cell viability curve was drawn with time point as abscissa and OD value as ordinate. The experiment was repeated three times.

### Clone formation experiment

Colon cancer cells in logarithmic growth phase were digested with 0.25% trypsin to prepare single cell suspension. After cell counting, 1000 cells were added into each well of a 12-well plate and shaken evenly. The cells were cultured in the 37 °C, 5% CO_2_ incubator, and the medium was changed every 2 days. When more than 50 cell clones were observed under microscope (about 7–14 days), the experiment was terminated. The cells were fixed with 4% polyformaldehyde for 15 min, and stained with 1% crystal violet (C0121; Beyotime Biotechnology Co., Shanghai, China) for 10 min. After the crystal violet staining solution was poured out, the staining solution of the cells was slowly washed away with running water and dried in the air. The 12-well plate were photographed under the microscope (Olympus, Tokyo, Japan) and the number of clones per well was counted. The experiment was repeated three times.

### Glucose uptake test

The colon cancer cells infected with sh-NC + oe-NC, sh-c-MYC + oe-NC, sh-c-MYC + oe-SULT2B1, sh-*OLR1* + oe-NC, or sh-*OLR1* + oe-SULT2B1 were added into the 6-well plate with 1 × 10^5^ cells per well. After 24 h in serum-free medium, cell culture medium was collected and centrifuged at room temperature for 5 min. After removing the precipitates, the supernatant was taken out. The blank and standard controls were set. According to the method of glucose determination kit (BC2505, Beijing Solarbio Science & Technology Co. Ltd.), enzyme colorimetry was used for determination. The working solution was prepared according to the ratio of R1 reagent to R2 reagent of 1:1, and used after mixing evenly. The 10 μL distilled water, calibration solution, and supernatant was added into 1000 μL working solution, respectively, to configure blank tube, calibration tube, and sample tube. After adding the sample, it was mixed well with vortex oscillator, placed in 37 °C constant temperature water bath, and reacted in water bath for 10 min. The absorbance value of the final product was detected at 505 nm by enzyme reader (BioTek, VT, USA). According to the instructions of the kit, the glucose level in the supernatant was calculated, which was the glucose uptake of the cells. The experiment was repeated three times.

### Lactic acid production test

According to the method of lactic acid determination kit (93-K627-100, BioVision, San Francisco, CA, USA), the standard curve was established with lactate standard. The mixture of 20 μL supernatant, 26 μL buffer, and 2 μL lactate enzyme was absorbed, mixed evenly, and placed at room temperature for 30 min. The absorbance of the final product at 570 nm was detected by microplate reader (BioTek). According to the standard curve, the level of lactic acid in the supernatant was calculated, which was the amount of lactic acid produced by cells. The experiment was repeated three times.

### Determination of ATP (adenosine triphosphate)/ADP (adenosine diphosphate) ratio by high-performance liquid chromatography (HPLC)

The colon cancer cells infected with sh-NC + oe-NC, sh-c-MYC + oe-NC, sh-c-MYC + oe-SULT2B1, sh-*OLR1* + oe-NC, or sh-*OLR1* + oe-SULT2B1 were cultured in 150 mL culture bottle with 1 × 10^6^ conventional cells. After 24 h of culture, the cell culture medium was collected. After centrifugation for 5 m, the cells were centrifuged repeatedly and rinsed once and counted. The cytomembrane was broken with 500 μL of 0.1 mmol/L perchloric acid. After centrifugation for 20 min, the supernatant was 250 μL. The 50 μL of 0.5 mmol/L Na_2_CO_3_ was added to mix. After centrifugation for 10 m, 50 μL supernatant was taken out to leave it for machine detection. HPLC detection method: ATP or ADP standard was taken to prepare 1 nmol/L solution. Afterwards, it added ddH_2_O to make a constant volume of 50 μL and chromatographic loading of 20 μL. The content of the sample was calculated by comparing the elution peak of the standard. ATP and ADP contents were expressed in nmol/10^6^ cells. Analysis conditions of HPLC: Waters 510 Pump Hypersil C18 (250 mm × 4.6 mm, the particle size of packing is 5 μM), column temperature 25 °C, the mobile phase was 0.1 mmol/L KH2P04 (pH 6.0), the injection volume was 20 μL, pump flow rate was 0.9 mL/min, constant speed elution, Waters 484 Photodiode Array Detector, the UV detection wavelength was 254 nm, Baseline 810 chromatographic workstation.

### Seahorse experiment

The Seahorse XF96 flux analyzer (Seahorse Bioscience, Billerica, MA, USA) was used to detect the extracellular acidification rate (ECAR) reflecting the activity of glycolytic metabolism. The cells were laid 24 h in advance. Colon cancer cells were seeded into the Seahorse XF cell culture plate at a density of 1 × 10^4^ per well and cultured overnight in growth medium. The instrument was preheated, seahorse instrument and computer were turned on, and software was turned on to make the instrument temperature rise to 37 °C with overnight preheating and hydration probe. The Seahorse XF calibration solution was added into the Utility Plate. The test plate was put back on the Utility Plate. And it was placed in a CO_2_-free incubator at 37 °C overnight to hydrate the probe. Drug concentration required for cell glycolysis pressure test (103020-100, Seahorse Bioscience): 10 mmol/L glucose, 1 mol/L oligomycin, and 50 mmol/L 2-deoxyglucose (2-DG). Mitochondrial stress test kit (103015-100, seahorse Bioscience), 1 mol/L oligomycin, 1 mol/L carbonyl cyanide 4-(trifluoromethoxy)phenylhydrazone (FCCP), 0.5 mol/L rotenone, and 0.5 mol/L actinomycin A. Both assays were normalized by total protein quantification. The experiment was repeated three times.

### Establishment and grouping of colon cancer xenograft model in nude mice

A total of 70 nude mice were purchased from the experimental animal center of the Third Military Medical University. The BALB/c nude mice aged 4 weeks and specific-pathogen-free (SPF) were fed on laminar flow shelves under the conditions of constant temperature (24–26 °C) and constant humidity (45–55%). Feed and drinking water were used after high-temperature disinfection.

The colon cancer cells in logarithmic growth phase were prepared into suspension containing 1 × 10^6^ colon cancer cells (200 μL). Iodophor solution was used to disinfect the skin of inoculation site. The 200 μL single cell suspension was subcutaneously injected into the left armpit of nude mice with a 1 mL syringe. After implantation, the nude mice were kept in SPF environment. Tumor nodules appeared in the inoculation site of nude mice about 2 weeks, and the texture was hard. When the tumor diameter was about 7 mm, it was considered that the nude mice model of colon cancer transplantation was successful.

Oxaliplatin intraperitoneal injection was performed [17]. The total dosage of oxaliplatin was calculated according to 30 mg/g body weight, and was given in five times. The dosage of each injection was 6 mg/kg, once every 3 days by intraperitoneal injection, five times in a row. Since the first administration, the maximum long diameter and vertical short diameter (mm) of tumor were measured by vernier caliper, which were expressed as *a* and *b*, respectively. The tumor volume (*V*) was calculated according to the following formula: *V* = *a* × *b*^2^ × 0.5 [18]. The tumor volume was measured every 5 days and four times in a row to draw tumor growth curve of tumor-bearing mice. The nude mice were euthanized after drug withdrawal one week. The tumor was dissected, weighed and the inhibition rate was calculated as [(average tumor weight of control group − average tumor weight of treatment group)/average tumor weight of control group × 100%] [18].

Nude mice were subcutaneously inoculated with colon cancer cells infected with sh-NC + oe-NC, sh-*OLR1* + oe-NC, or sh-*OLR1* + oe-SULT2B1; or [2] subcutaneously inoculated with colon cancer cells infected with sh-NC or sh-*OLR1* and then subcutaneously injected with PBS or oxaliplatin (*n* = 10 mice/group).

### Statistical analysis

SPSS21.0 (IBM-SPSS Inc., Chicago, IL, USA) was implemented for statistical analysis. The measurement data were expressed in the form of mean ± standard deviation. The enumeration data were indicated as a rate or percentage. The *χ2* test was used in the comparison of enumeration data. Independent sample *t*-test was adopted for comparison between the two groups. One-way analysis of variance (ANOVA) was applied for comparison among multiple groups. Kaplan-Meier survival method and Log-rank test were used to analyze the relationship between expression intensity of *OLR1* and survival time of patients with colon cancer. *p* < 0.05 was considered to be statistically significant difference.

## Results

### *OLR1* is negatively correlated with the survival of patients with colon cancer

**Fig. 1:**
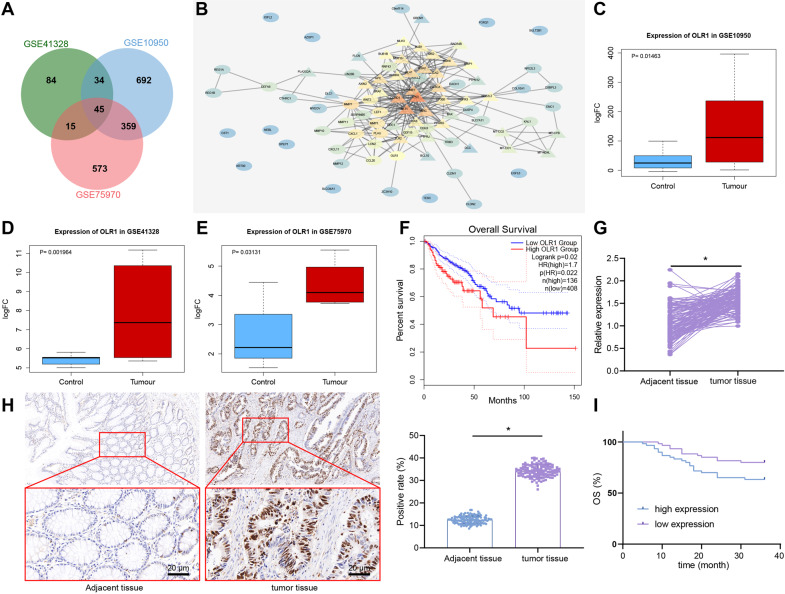
*OLR1* expression is high in colon cancer tissues. **A** Venn map of upregulated genes in GSE10950, GSE41328, and GSE75970, which obtained 45 overlapping genes. **B** Protein interaction network of 45 overlapping genes and 50 important genes of colon cancer from MalaCards. Circles represented intersecting genes and triangles represented genes obtained by MalaCards. The redder the color of the gene image, the higher the degree of core. Conversely, the bluer the color, the lower the degree of core. **C** The box diagram of the expression of *OLR1* in GSE10950 (control *n* = 24; tumor *n* = 24). **D** The box diagram of the expression of *OLR1* in GSE41328 (control *n* = 24; tumor *n* = 24). **E** The box diagram of the expression of *OLR1* in GSE75970 (control *n* = 24; tumor *n* = 24). The blue box indicated the expression of normal samples, and the red box indicated the expression of tumor samples. **F** GEPIA to analyze TCGA clinical data to draw the curve between *OLR1* expression and colon cancer survival. **G** RT-qPCR to detect the expression of *OLR1* mRNA in colon cancer tissues and adjacent normal tissues (adjacent tissues: *n* = 120; tumor tissues: *n* = 120). **H** The expression of LOX-1 protein in colon cancer tissues and adjacent normal tissues detected by immunohistochemistry (adjacent tissues: *n* = 120; tumor tissues: *n* = 120; ×500). **I** Kaplan-Meier curve of the relationship between *OLR1* and survival in patients with colon cancer. *p < 0.05 between two groups.

In order to find the targets for the treatment and diagnosis of colon cancer, 1130, 178, and 992 upregulated genes in colon cancer were found by differential analysis of expression datasets GSE10950, GSE41328, and GSE75970, respectively, which obtained 45 genes from their intersection (Fig. 1A). The 50 colon cancer-related genes were obtained from MalaCards, and the protein–protein interaction network of these 95 genes was constructed using String (Fig. 1B) to calculate the core degree of 45 significantly upregulated genes. Thus, the top 10 genes were retrieved, among which the research on *OLR1* was the least. Moreover, *OLR1* was highly expressed in colon cancer in these three datasets (Fig. 1C–E). The results suggested that *OLR1* might be involved in the occurrence and development of colon cancer.

In order to further determine the role of *OLR1* in colon cancer, the clinical data (COADREAD) of colon cancer in the TCGA were analyzed by GEPIA. The results displayed that the survival rate of colon cancer patients with high expression of *OLR1* was strikingly decreased (Fig. 1F). As reflected by RT-qPCR and immunohistochemical staining in 120 cases of colon cancer tissues and adjacent normal tissues, *OLR1* expression was potently higher in colon cancer tissues than that in adjacent normal tissues, and LOX-1 protein was mainly located in the cell membrane of colon cancer tissues (Fig. 1G, H).

As documented in Supplementary Table 2, high expression of *OLR1* in colon cancer tissues had sharp positive correlation with lymph node metastasis and tumor infiltration depth of colon cancer patients, whereas the relative expression of *OLR1* was not correlated with age, gender, tumor size, tumor differentiation degree, and tumor location of patients. Based on the results of Kaplan-Meier, the overall survival of colon cancer patients with *OLR1* low expression was dramatically longer than that in patients with high expression (Fig. 1I), which was consistent with the biological prediction. In brief, *OLR1* was highly expressed in colon cancer tissues, and was negatively correlated with survival time of colon cancer patients, and positively correlated with lymph node metastasis and tumor infiltration depth of colon cancer patients.

### Knockdown of *OLR1* downregulates c-MYC and inhibits the proliferation and chemoresistance of colon cancer cells

**Fig. 2:**
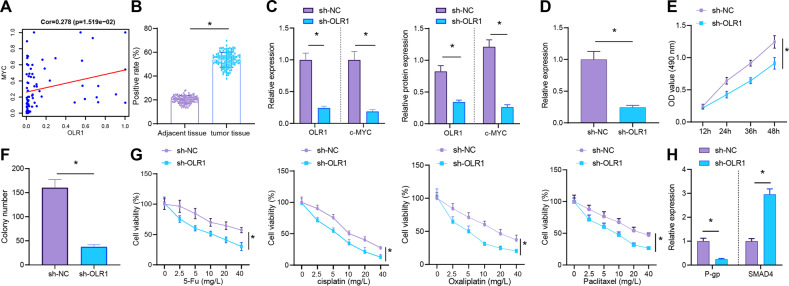
*OLR1* knockdown depresses c-MYC expression and the proliferation and chemoresistance of colon cancer cells. **A** Correlation map of expression data of *OLR1* and c-MYC in GSE10950, GSE41328, and GSE75970. **B** Immunohistochemical staining to detect the expression of c-MYC protein in colon cancer tissues and adjacent normal tissues (adjacent tissues: *n* = 120; tumor tissues: *n* = 120). **C** RT-qPCR and western blot analysis to detect the expression of *OLR1* and c-MYC in colon cancer cells. **D** Detection of Ki67 mRNA expression in colon cancer cells by RT-qPCR. **E** MTS to detect the proliferation of colon cancer cells. **F** Colony formation assay to detect the colony formation of colon cancer cells. **G** The drug resistance of colon cancer cells to DDP, oxaliplatin, 5-Fu, and paclitaxel detected by MTS. **H** RT-qPCR to detect the mRNA expression of P-gp and SMAD4 in colon cancer cells. The cell experiment was repeated three times. *p < 0.05 between two groups.

In order to further determine the relationship between *OLR1* and c-MYC in colon cancer, the expression data of *OLR1* and c-MYC in GSE10950, GSE41328, and GSE75970 were normalized, respectively. Then the expression correlation between *OLR1* and c-MYC was analyzed by merging these three datasets. The results showed that *OLR1* expression was positively correlated with c-MYC in colon cancer (Fig. 2A), indicting *OLR1* might promote c-MYC expression in colon cancer. Immunohistochemistry authenticated that compared with adjacent normal tissues, c-MYC was substantially upregulated in colon cancer tissues, which was mainly located in the nucleus of colon cancer tissues (Fig. 2B).

The *OLR1* stably knockdown cell lines and control cell lines were constructed to explore the regulation of c-MYC expression by *OLR1* in colon cancer cells and its effect on the function of colon cancer cells. As for the result of RT-qPCR and western blot analysis, knockdown of *OLR1* evidently reduced the transcription level of c-MYC (Fig. 2C). In addition, according to RT-qPCR result, knockdown of *OLR1* strikingly disturbed proliferation marker Ki67 expression (Fig. 2D). Meanwhile, MTS test and clone formation test exhibited knockdown of *OLR1* remarkably decreased the proliferation and clone formation of colon cancer cells (Fig. 2E, F).

The sensitivity of LoVo colon cancer cells to chemotherapeutic drugs and P-gp and SMAD4 expression [19] were detected before and after the knockdown of *OLR1*. In the experiment of cell drug resistance, the conventional treatment drugs such as DDP, oxaliplatin, 5-Fu, and paclitaxel were selected for the study [16]. The results documented that the sensitivity of LoVo colon cancer cells to DDP, oxaliplatin, 5-Fu, and paclitaxel was obviously increased, P-gp expression was significantly decreased, and SMAD4 expression was clearly enhanced after knocking down *OLR1* (Fig. 2G, H). In conclusion, high expression of c-MYC in colon cancer tissues and cells was observed, and knockdown of *OLR1* could downregulate c-MYC and repress the proliferation and chemoresistance of colon cancer cells.

### Knockdown *OLR1* suppresses the proliferation and chemoresistance of colon cancer cells by downregulating c-MYC

**Fig. 3:**
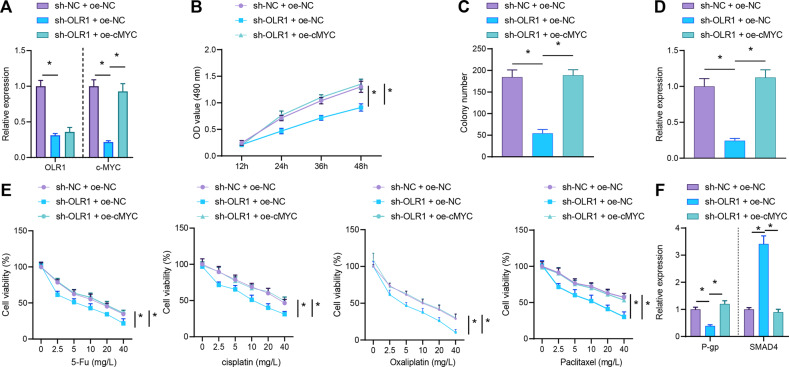
*OLR1* knockdown causes c-MYC downregulation to restrain proliferation and chemoresistance of colon cancer cells. Colon cancer cells were treated with sh-NC + oe-NC, sh-*OLR1* + oe-NC, or sh-*OLR1* + oe-c-MYC. **A** The mRNA expression of *OLR1* and c-MYC in colon cancer cells determined by RT-qPCR. **B** MTS to measure the proliferation of colon cancer cells. **C** Colony formation of colon cancer cells detected by colony formation assay. **D** The mRNA expression of Ki67 in colon cancer cells checked by RT-qPCR. Colon cancer cells were treated with sh-NC + oe-NC, sh-*OLR1* + oe-NC, or sh-*OLR1* + oe-c-MYC, followed by treatment with DDP, oxaliplatin, 5-Fu, and paclitaxel. **E** The drug resistance of colon cancer cells to DDP, oxaliplatin, 5-Fu, and paclitaxel assessed by MTS. **F** RT-qPCR to detect the mRNA expression of P-gp and SMAD4 in colon cancer cells. The cell experiments were repeated three times. *p < 0.05 between two groups.

In order to further verify that *OLR1* regulated the proliferation and chemoresistance of colon cancer cells by promoting c-MYC, *OLR1* was knocked down and/or c-MYC was overexpressed in colon cancer cells. From RT-qPCR result, *OLR1* and c-MYC mRNA expression was obviously diminished in *OLR1*-silenced colon cancer cells, while in the presence of sh-*OLR1*, elevated c-MYC mRNA expression was observed in colon cancer cells after further oe-c-MYC treatment (Fig. 3A). As detected by MTS assay, colony formation assay, and RT-qPCR, the proliferation, colony formation, and Ki67 expression of LoVo colon cancer cells was apparently decreased by sh-*OLR1* treatment, which was abrogated by further oe-c-MYC treatment (Fig. 3B–D). Furthermore, the sensitivity of LoVo colon cancer cells to DDP, oxaliplatin, 5-Fu, and paclitaxel was distinctly enhanced, P-gp expression was authentically declined, and SMAD4 expression was noticeably augmented subsequent to *OLR1* knockdown, which was reversed by further overexpressing c-MYC (Fig. 3E, F). Collectively, knockdown of *OLR1* could inhibit the proliferation and chemoresistance of colon cancer cells via c-MYC downregulation.

### Knockdown of c-MYC reduces SULT2B1 expression and inhibits the glycolytic metabolism of colon cancer cells

**Fig. 4:**
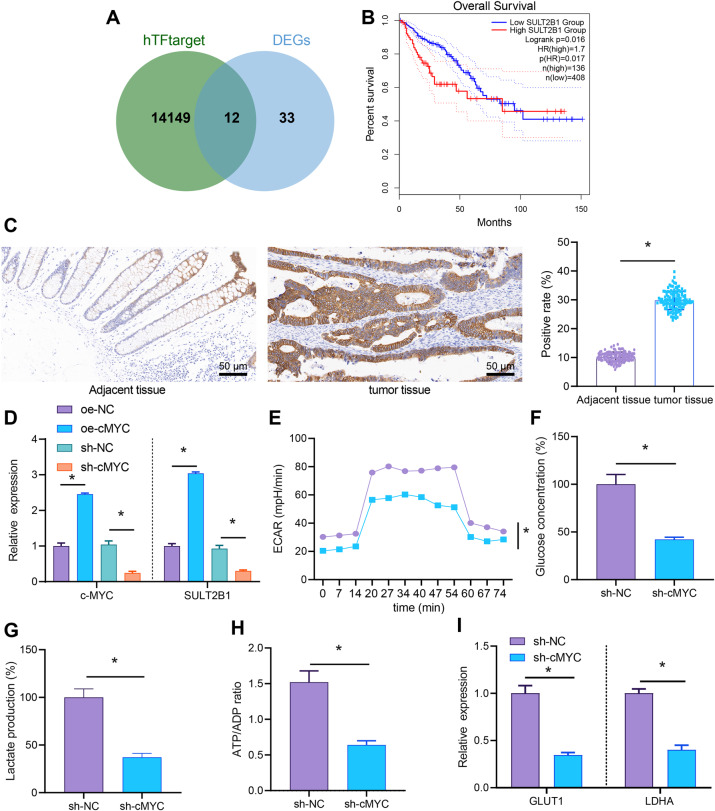
c-MYC knockdown diminishes SULT2B1 expression and dampened glycolytic metabolism of colon cancer cells. **A** The downstream genes of transcription factor c-MYC predicted by hTFtarget and intersected with 45 obviously upregulated genes to obtain 12 genes. **B** Clinical data of TCGA analyzed by GEPIA to draw the survival curve between SULT2B1 expression and colon cancer. **C** The protein expression of c-MYC in colon cancer and adjacent normal tissues detected by immunohistochemistry (adjacent tissues: *n* = 120; tumor tissues: *n* = 120; ×200). **D** The mRNA expression of c-MYC and SULT2B1 in colon cancer cells after overexpression and knocking down c-MYC determined by RT-qPCR. **E** Detection of ECAR in colon cancer cells after knocking down c-MYC. **F** Detection of glucose content in colon cancer cells after knocking down c-MYC. **G** Detection of lactate production in colon cancer cells after knocking down c-MYC. **H** Detection of ATP/ADP in colon cancer cells after knocking down c-MYC. **I** RT-qPCR to detect the mRNA expression of GLUT1 and LDHA in colon cancer cells after knocking down c-MYC. The cell experiment was repeated three times. *p < 0.05 between two groups.

Next, totally 14,161 downstream genes of c-MYC were predicted by hTFtarget and intersected with 45 significantly upregulated genes, which found that 12 genes were critical downstream genes of c-MYC (Fig. 4A). By analyzing the clinical data (COADREAD) of colon cancer in TCGA database through GEPIA, the relationship between these 12 genes and survival of colon cancer was obtained, which found that only SULT2B1 and GDF15 were certainly correlated with survival, and the correlation of SULT2B1 was significantly higher than that of GDF15 (Supplementary Table 3 and Fig. 4B). Therefore, we believed that SULT2B1 might be a key downstream gene of c-MYC in colon cancer. The results of immunohistochemical staining exhibited that compared with the adjacent normal tissues, SULT2B1 was highly expressed in colon cancer tissues and mainly located in the cytoplasm of colon cancer tissues (Fig. 4C).

Furthermore, the colon cancer cells were infected with lentivirus to confirm that c-MYC could orchestrate SULT2B1 expression. As reflected by RT-qPCR, increased c-MYC and SULT2B1 expression in LoVo colon cancer cells was noticed following c-MYC overexpression, which was opposite after silencing c-MYC (Fig. 4D), indicating that c-MYC could upregulate SULT2B1.

It was found that SULT2B1 was related to the steroid hormone biosynthesis (hsa00140) pathway using KEGG database [20]. Through literature review, we found that steroid hormones could mediate glycolytic metabolism that could impact cell proliferation and chemoresistance of various cancers [21–25]. Therefore, ECAR, glucose uptake, lactate production, ATP/ADP ratio, and the expression of glycolysis-related genes GLUT1 and LDHA [26] were detected. On the basis of detection result, the ECAR, glucose intake, lactate production, ATP/ADP ratio, and GLUT1 and LDHA expression of LoVo colon cancer were apparently diminished by knocking down c-MYC (Fig. 4E–I). The results suggested that knockdown of c-MYC could inhibit glycolytic metabolism of colon cancer cells. Taken together, SULT2B1 was highly expressed in colon cancer cells, and knockdown of c-MYC could disturb SULT2B1 expression and inhibit the glycolytic metabolism of colon cancer cells.

### Knockdown of c-MYC inhibits the proliferation and chemoresistance of colon cancer cells by decreasing SULT2B1 expression to restrain glycolytic metabolism

**Fig. 5:**
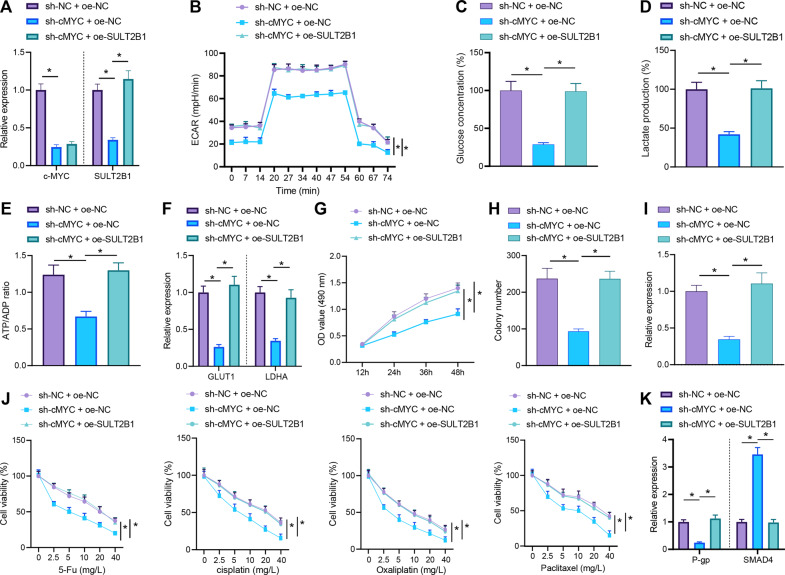
Inactivation of c-MYC/SULT2B1 axis disturbs glycolytic metabolism to diminish proliferation and chemoresistance of colon cancer cells. Colon cancer cells were treated with sh-NC + oe-NC, sh-c-MYC + oe-NC, or sh-c-MYC + oe-SULT2B1. **A** The mRNA expression of c-MYC and SULT2B1 in colon cancer cells tested by RT-qPCR. **B** Detection of ECAR in colon cancer cells. **C** Glucose content in colon cancer cells. **D** Lactate production in colon cancer cells. **E** The ATP/ADP in colon cancer cells. **F** RT-qPCR to measure the mRNA expression of GLUT1 and LDHA in colon cancer cells. **G** MTS to calculate the proliferation level of colon cancer cells. **H** Colony formation assay to check the level of colony formation in colon cancer cells. **I** Detection of Ki67 mRNA expression in colon cancer cells by RT-qPCR. Colon cancer cells were treated with sh-NC + oe-NC, sh-c-MYC + oe-NC, or sh-c-MYC + oe-SULT2B1, followed by treatment with DDP, oxaliplatin, 5-Fu, and paclitaxel. **J** The drug resistance of colon cancer cells to DDP, oxaliplatin, 5-Fu, and paclitaxel detected by MTS. **K** RT-qPCR for detecting the mRNA expression of P-gp and SMAD4 in colon cancer cells. The cell experiments were repeated three times. *p < 0.05 between two groups.

In order to further explore whether c-MYC manipulated glycolytic metabolism through SULT2B1 to affect the proliferation and chemoresistance of colon cancer cells, we successfully constructed a cell line with c-MYC knockdown and SULT2B1 overexpression (Fig. 5A). The ECAR, glucose uptake, lactate production, ATP/ADP ratio, and GLUT1 and LDHA (glycolysis-related genes) expression were detected, which displayed that overexpression of SULT2B1 restored the inhibition of glycolysis induced by c-MYC knockdown (Fig. 5B–F). Meanwhile, MTS assay, clone formation assay, and RT-qPCR manifested that overexpression of SULT2B1 negated the repressive effect of c-MYC knockdown on colon cancer cell proliferation (Fig. 5G–I).

In addition, control cells (the sh-NC + oe-NC group), c-MYC knockdown cells (the sh-c-MYC + oe-NC group), and c-MYC knockdown cells with overexpression of SULT2B1 (the sh-c-MYC + oe-SULT2B1 group) were stimulated with chemotherapeutic drugs including DDP, oxaliplatin, 5-Fu, and paclitaxel. Overexpression of SULT2B1 neutralized the sensitization effect of colon cancer cells to chemotherapeutic drugs induced by c-MYC knockdown (Fig. 5J, K). In short, knockdown of c-MYC could decline SULT2B1 expression to curtailed glycolytic metabolism, thereby inhibiting the proliferation and chemoresistance of colon cancer cells.

### Knockdown of *OLR1* suppresses glycolytic metabolism by downregulating c-MYC/SULT2B1 axis to restrain the proliferation and chemoresistance of colon cancer cells

**Fig. 6:**
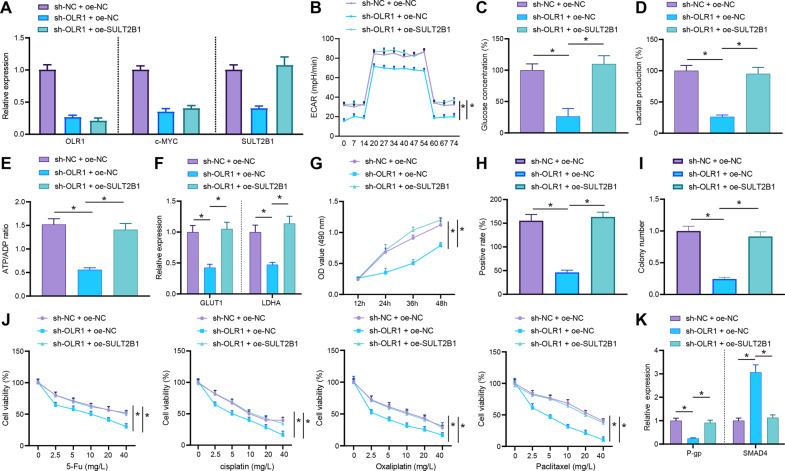
Blockade of *OLR1*/c-MYC/SULT2B1 axis reduces glycolytic metabolism to restrain proliferation and chemoresistance of colon cancer cells. Colon cancer cells were transfected with sh-NC + oe-NC, sh-*OLR1* + oe-NC, or sh-*OLR1* + oe-SULT2B1. **A** The mRNA expression of *OLR1*, c-MYC, and SULT2B1 in colon cancer cells checked by RT-qPCR. **B** ECAR of colon cancer cells. **C** Glucose content in colon cancer cells. **D** Lactate production in colon cancer cells. **E** The ATP/ADP in colon cancer cells. **F** RT-qPCR to detect the mRNA expression of GLUT1 and LDHA in colon cancer cells. **G** The proliferation level of colon cancer cells estimated using MTS. **H** Detection of colon cancer cell clone formation. **I** The mRNA expression of Ki67 detected by RT-qPCR. Colon cancer cells were transfected with sh-NC + oe-NC, sh-*OLR1* + oe-NC, or sh-*OLR1* + oe-SULT2B1, followed by treatment with DDP, oxaliplatin, 5-Fu, and paclitaxel. **J** The drug resistance of colon cancer cells to DDP, oxaliplatin, 5-Fu, and paclitaxel assessed by MTS. **K** The expression of P-gp and SMAD4 in colon cancer cells tested by RT-qPCR. The cell experiment was repeated three times. *p < 0.05 between two groups.

Based on the above results, we speculated that *OLR1* could activate the transcription of SULT2B1 by promoting c-MYC to enhance glycolytic metabolism, cell proliferation, and chemotherapy resistance. A cell line with *OLR1* knockdown and SULT2B1 overexpression was successfully constructed to further verify this conjecture (Fig. 6A). ECAR, glucose uptake, lactate production, ATP/ADP ratio, and GLUT1 and LDHA expression were evaluated, which depicted that overexpression of SULT2B1 nullified the repression of glycolysis induced by *OLR1* knockdown (Fig. 6B–F).

The MTS assay, clone formation assay, and Ki67 expression also manifested that overexpression of SULT2B1 annulled the suppression of the proliferation of colon cancer cells triggered by *OLR1* knockdown (Fig. 6G–I). Colon cancer cells were stimulated by chemotherapeutic drugs (DDP, oxaliplatin, 5-Fu, and paclitaxel). It was observed that overexpression of SULT2B1 counteracted the sensitization effect of *OLR1* knockdown to chemotherapy drugs (Fig. 6J, K). All in all, knockdown of *OLR1* might downregulate SULT2B1 to repress glycolytic metabolism, thereby dampening the proliferation and chemoresistance of colon cancer cells.

### Knockdown of *OLR1* blocks growth and chemoresistance of colon cancer in nude mice by downregulating c-MYC/SULT2B1

**Fig. 7:**
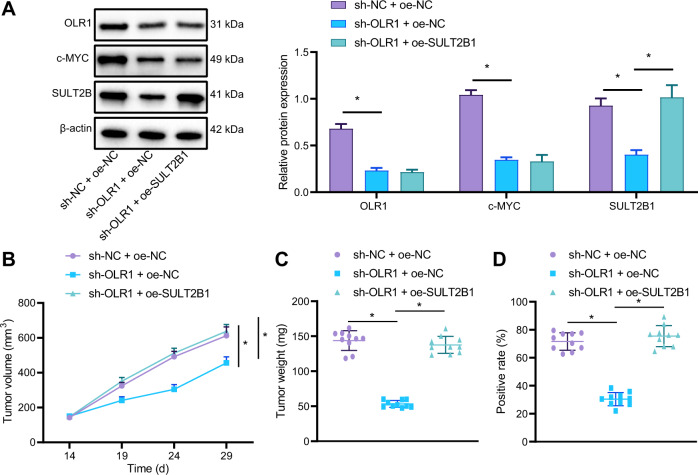
The inactivation of *OLR1*/c-MYC/SULT2B1 axis restrains tumorigenicity and chemoresistance of colon cancer cells in nude mice. **A** The protein expression of LOX-1, c-MYC, and SULT2B1 detected by western blot analysis. **B** Growth curve of subcutaneous colon cancer xenograft in nude mice. **C** Weight of tumor in nude mice. **D** The expression of Ki67 in tumor tissues of nude mice detected by immunohistochemistry (*n* = 10 mice/group). *p < 0.05.

In order to further explore whether *OLR1*/c-MYC/SULT2B1 axis could also modulate the growth and chemoresistance of colon cancer cells in vivo, a subcutaneous tumor transplantation experiment was implemented in nude mice. The LoVo cells transfected with sh-NC + oe-NC, sh-*OLR1* + oe-NC, or sh-*OLR1* + oe-SULT2B1 were subcutaneously injected into nude mice separately. As indicated by western blot analysis, expression of LOX-1, c-MYC, and SULT2B1 in tumor tissues of nude mice was lowered by knocking down *OLR1*, whilst SULT2B1 expression in tumor tissues of nude mice was markedly strengthened by oe-SULT2B1 treatment in the presence of sh-*OLR1*As (Fig. 7A). Tumor growth rate and weight, and Ki67 expression were reduced in nude mice by sh-*OLR1* treatment, which was negated by further oe-SULT2B1 treatment (Fig. 7B–D). These results suggested that knockdown of *OLR1* slowed down the growth of colon cancer cells in nude mice via downregulation of SULT2B1.

The cells treated with sh-NC or sh-*OLR1* were injected subcutaneously into nude mice to establish nude mice model of colon cancer. After intraperitoneal injection with drugs or normal saline, the tumor inhibition rate was detected. The result displayed that in the presence of oxaliplatin, the tumor inhibition rate was augmented by sh-*OLR1* (Supplementary Table 4). Generally speaking, knockdown of *OLR1* might inhibit the tumorigenicity and chemoresistance of colon cancer cells in nude mice by downregulating c-MYC and SULT2B1.

## Discussion

**Fig. 8:**
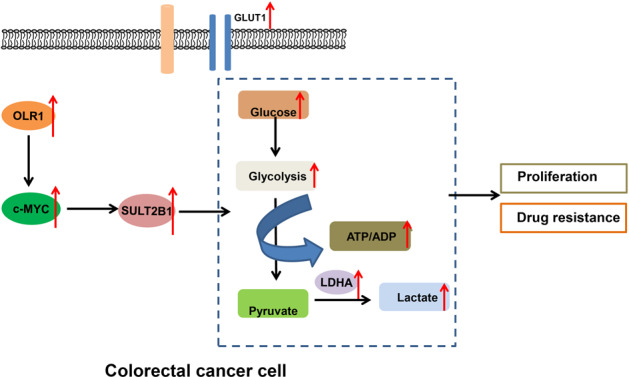
Mechanism graph of the regulatory network and function of *OLR1*. *OLR1* promotes colon cancer cell proliferation and chemoresistance by regulating c-MYC/SULT2B1 axis to enhance glycolysis metabolism.

Patients with colon cancer are commonly diagnosed at regional or distant metastatic stage, which creates the need of adjuvant chemotherapy after their surgery or palliative chemotherapy for their metastatic disease [27]. However, the stemness and expansion abilities of colon cancer cells can result in resistance to conventional chemotherapies, which impacts the therapeutic effect of chemotherapy on patients with colon cancer [28]. Therefore, there exists an ongoing need to research the molecular mechanism behind the chemoresistance of colon cancer. In this context, we conducted this research to investigate the mechanism of *OLR1*/c-MYC/SULT2B1 axis in chemoresistance of colon cancer, and uncovered that knockdown of *OLR1* could downregulate c-MYC to diminish the transcription of SULT2B1, thus repressing glycolytic metabolism and then reducing the proliferation and chemoresistance of colon cancer cells (Fig. 8).

In the current work, evidence was obtained demonstrating that *OLR1* was expressed at a high level in colon cancer tissues, shared negative correlation with the survival of patients with colon cancer, and facilitated glycolytic metabolism to promote proliferation and chemoresistance of colon cancer cells. A similar trend observed in the data of a prior research that *OLR1* upregulation accelerated colon cancer onset, progression, and metastasis [8]. Moreover, Hirsch et al. has found that OLR1 is involved in maintaining the transformed state in developmentally diverse cancer cell lines and in tumor growth [29]. Of note, LOX-1 silencing contributes to impairment in proliferation rate, closing the scratch, growth, and tumorigenicity of CRC cells [30]. More importantly, a research conducted by Nakashima-Nakasuga et al. elucidated that CRC patients with high *OLR1* expression in tumor tissues showed obviously poorer prognosis than individuals with low expression [31]. Moreover, the overexpression of *OLR1* has been detected in breast cancer tissues, and enhanced *OLR1* expression is able to increase breast cancer cell proliferation [32]. Besides, a prior study noted that *OLR1* presented with high-expression in osteosarcoma tissues and that osteosarcoma cell proliferation was accelerated when *OLR1* was ectopically expressed [33]. It is widely reported that cancer cells commonly have augmented glycolytic metabolism for ATP generation, which frequently correlates to drug resistance in cancers [34]. Intriguingly, a prior work manifested that the induction of glycolytic metabolism facilitated chemoresistance in CRC [35]. Meanwhile, *OLR1* has been identified to assume a crucial role in glycolytic metabolism [36]. Consistently, *OLR1* upregulation was observed in pancreatic cancer tissues and was associated with the poor prognosis of patients, and then *OLR1* abrogation caused resistance of pancreatic cancer cells to gemcitabine [9]. Here, we propose that *OLR1* may participate in glycolytic metabolism, proliferation, and chemoresistance of colon cancer cells. In addition, our in vivo experiments manifested that OLR1 silencing diminished tumor weight and volume, Ki67 expression, and tumor chemoresistance in nude mice. Similarly, a prior research demonstrated that decreased Ki67 expression was correlated with reduced proliferation of CRC cells in vivo [37]. Of note, another research displayed that the inhibition of *OLR1* could prevent the tumorigenesis and metastasis formation of CRC in xenograft tumors of nude mice, suggesting *OLR1* as a promising target for suppression of tumor progression and metastasis of CRC [38]. Partially concordant with our in vivo findings, the research of Xiong et al. ascertained that OLR1 upregulation caused by GSTM3TV2 was capable of augmenting chemoresistance in nude mice with subcutaneously injected pancreatic cancer cells and treated with gemcitabine [9].

As previously documented, *OLR1* triggered c-MYC upregulation to accelerate pancreatic cancer metastasis [10]. A similar finding was noted in our work that *OLR1* knockdown diminished c-MYC expression to suppress colon cancer cell glycolytic metabolism, proliferation, and chemoresistance. Corroborating findings were reported in a previous research that c-MYC was upregulated in CD133 colon cancer stem cells, and that upregulated c-MYC caused cell proliferation and chemoresistance of colon cancer [39]. In addition, c-MYC downregulation was reported to lower the proliferation and reduce glucose consumption, lactate production, and ATP production to depress glycolytic metabolism in DLD-1 and SW480 colon cancer cells, which was concordant with our results [40]. Also, stabled c-MYC was capable of contributing to CRC cell proliferation by augmenting glycolytic metabolism [26]. However, the mechanism of c-MYC in glycolytic metabolism and chemoresistance in colon cancer remained to be further investigated. The obtained data in our research manifested that knockdown of c-MYC depressed the proliferation and chemoresistance of colon cancer cells by diminishing glycolytic metabolism via SULT2B1 downregulation. Concurrent with our results, a research conducted by Li et al. elaborated that SULT2B1 overexpression was linked to the development of CRC [13]. More importantly, SULT2B1 silencing caused disturbed HCC cell proliferation, which is partially in consent with our finding [41].

To sum up, the conclusion of the current study was that *OLR1* knockdown disrupted the activation of c-MYC/SULT2B1 axis to repress glycolytic metabolism, thus restraining cell proliferation and chemoresistance in colon cancer. The result could open a novel candidate target in the field of colon cancer treatment. However, there exists limited research about the role of SULT2B1 in glycolytic metabolism and chemoresistance. This background calls for further research to figure out whether SULT2B1 manipulated glycolytic metabolism and chemoresistance in other tumors and find more convincible data of our study.

## Supplementary information

Supplementary Tables.

## Data Availability

The datasets generated/analyzed during the current study are available from the corresponding author on reasonable request.
