# Supplementary material for: Knockdown of OLR1 weakens glycolytic metabolism to repress colon cancer cell proliferation and chemoresistance by downregulating SULT2B1 via c-MYC
Source: Cell Death Dis. 2021 Dec 17;13(1):4. doi: 10.1038/s41419-021-04174-w (PMC8683511; doi:10.1038/s41419-021-04174-w)
Supplement: Supplementary file 1 — Supplementary Tables. [file 41419_2021_4174_MOESM1_ESM.docx]

**Supplementary Table 1** Primers for RT-qPCR

| *OLR1* | F: 5’- CTTTGGATGCCAAGTTGCTGAA -3’ |
| --- | --- |
|  | R: 5’- GCATCAAAGGAGAACCGTCC -3’ |
| c-MYC | F: 5’- TAGTGGAAAACCAGCAGCCTC -3’ |
|  | R: 5’- AAGTTCTCCTCCTCGTCGCA -3’ |
| SULT2B1 | F: 5’- CCCACCTCCCTATTGAACTG -3’ |
|  | R: 5’- CGGCCCAAGTAAATCACCT -3’ |
| Ki67 | F: 5’- TCCTTTGGTGGGCACCTAAGACCTG -3’ |
|  | R: 5’- TGATGGTTGAGGTCGTTCCTTGATG -3’ |
| GLUT1 | F: 5’- TATCTGAGCATCGTGGCCAT -3’ |
|  | R: 5’- AAGACGTAGGGACCACACAG-3’ |
| LDHA | F: 5’- TGTCTCCAGCAAAGACTACTGT -3’ |
|  | R: 5’- GACTGTACTTGACAATGTTGGGA -3’ |
| P-gp | F: 5’- CACCTGGACGTTACCAAAGAAGATATA -3’ |
|  | R: 5’-TCACCAACCAGCGTCTCATATTT -3’ |
| SMAD4 | F: 5'- GCTGCTGGAATTGGTGTTGATG -3' |
|  | R: 5'- AGGTGTTTCTTTGATGCTCTGTCT -3' |
| GAPDH | F: 5’- GGAGCGAGATCCCTCCAAAAT -3’ |
|  | R: 5’- GGCTGTTGTCATACTTCTCATGG -3’ |

**Supplementary Table 2** Relationship between *OLR1* expression and clinical features of patients with colon cancer

| Group | n = 120 | *OLR1* mRNA relative expression | |
| --- | --- | --- | --- |
|  |  | 2^-△ct^ | *p* |
| Age | | | |
| ≥ 60 old | 66 | 1.61 ± 0.26 | > 0.05 |
| < 60 old | 54 | 1.57 ± 0.24 |  |
| Gender | | | |
| male | 82 | 1.60 ± 0.25 | > 0.05 |
| female | 38 | 1.57 ± 0.24 |  |
| Tumor size | | | |
| ≥ 5cm | 31 | 1.57 ± 0.25 | > 0.05 |
| < 5cm | 89 | 1.60 ± 0.25 |  |
| Differentiation degree | | | |
| High, medium | 77 | 1.58 ± 0.25 | > 0.05 |
| low | 43 | 1.60 ± 0.24 |  |
| Infiltration depth | | | |
| T_1, 2_ | 69 | 1.51 ± 0.25 | < 0.05 |
| T_3, 4_ | 51 | 1.69 ± 0.21 |  |
| Lymph node metastasis | | | |
| Yes | 65 | 1.65 ± 0.25 | < 0.05 |
| No | 55 | 1.52 ± 0.23 |  |
| Tumor location | | | |
| Ascending colon | 42 | 1.61 ± 0.27 | > 0.05 |
| Transverse colon | 11 | 1.51 ± 0.28 |  |
| Descending colon | 29 | 1.67 ± 0.21 |  |
| Sigmoid colon | 38 | 1.52 ± 0.22 |  |

**Supplementary Table 3** Survival analysis of 12 important downstream genes of c-MYC in colon cancer

| Gene | *p*.Value | HR |
| --- | --- | --- |
| SULT2B1* | 0.016 | 1.7 |
| GDF15* | 0.018 | 0.5 |
| NFE2L3 | 0.098 | 0.62 |
| OSBPL3 | 0.21 | 1.3 |
| RNF43 | 0.24 | 0.71 |
| DPEP1 | 0.38 | 0.79 |
| MMP11 | 0.45 | 1.2 |
| TRIB3 | 0.47 | 1.2 |
| MYEOV | 0.6 | 1.1 |
| SLC7A11 | 0.63 | 0.88 |
| SLCO4A1 | 0.68 | 1.1 |
| ENC1 | 0.79 | 1.1 |

**Notes:** *p.* Value represented a significant association between genes and survival. HR (hazard ratio) was the risk of gene expression relative to survival rate. * *p* < 0.05 indicated the expression gene had significant effect on the survival rate of colon cancer.

**Supplementary Table 4** Comparison of tumor weight and tumor inhibition rate in nude mice of each group

| Group | N | | Tumor weight (mg) | Tumor inhibition |
| --- | --- | --- | --- | --- |
|  | Before | After |  | Rate (%) |
| PBS + sh-NC | 10 | 10 | 128.84 ± 7.12 | - |
| PBS + sh-*OLR1* | 10 | 10 | 101.88 ± 6.39^*^ | 30 |
| Oxaliplatin + sh-NC | 10 | 10 | 100.91 ± 6.96 | 30 |
| Oxaliplatin + sh-*OLR1* | 10 | 10 | 71.61 ± 5.11^#^ | 60 |

Notes: * *p* < 0.05 compared with mice treated with PBS + sh-NC. # *p* < 0.05 compared with mice treated with Oxaliplatin + sh-NC.
